# Determinants of Stillbirth Among Deliveries Attended at Public Hospitals of Southwest Shoa Zone, Oromia, Ethiopia: A Case‐Control Study

**DOI:** 10.1002/hsr2.70806

**Published:** 2025-04-29

**Authors:** Bereket Abebe Alayu, EliasTeferi Balla, Daniel Belema Fekene, Galana Takele Namara, Marta Fekadu

**Affiliations:** ^1^ Oromia Health Bureau Addis Ababa Ethiopia; ^2^ College of Medicine and Health Science Ambo University Ambo Ethiopia; ^3^ Alert Hospital Addis Ababa Ethiopia

**Keywords:** antepartum, determinants, public hospital, Southwest Shoa, stillbirth

## Abstract

**Background:**

During the past 20 years, there have been 48 million stillbirths worldwide. One of the most common adverse pregnancy outcomes in developing nations, such as Ethiopia, is stillbirth.

**Aim:**

The aim of this study was to determine the factors that contribute to stillbirths among babies born in Ethiopia's Southwest Shoa Zone.

**Methods:**

Three hundred and two research participants were included in the hospital‐based unmatched case‐control study. The data extraction checklist was used to collect data from the medical records. To identify the determinants of stillbirth, bivariate and multivariate logistic regression analyses were used. The determinants of the stillbirth were identified in the multivariate logistic regression analysis with a *p*‐value of less than 0.05% and 95% CI.

**Results:**

A total of 302 maternal charts were reviewed, yielding a 100% response rate. Pre‐eclampsia/eclampsia (AOR: 3.3, 95% CI: 1.2–9.2), antepartum hemorrhage (AOR: 5.8, 95% CI: 2.1–15.9), a referral from other health facilities (AOR: 2.8, 95% CI: 1.4–5.3), obstructed labor (AOR: 3.8, 95% CI: 1.9–7.8), duration of labor > 24 h. (AOR: 4.7, 95% CI: 2.3–9.6), gestational age < 37 weeks (AOR: 3.5, 95% CI: 1.7–7.4) and congenital defect (AOR: 6.9, 95% CI: 2.4–19.7) were determinants of still birth.

**Conclusion:**

This study highlights that obstetric complications, gestational age, congenital abnormalities, and low birth weight were determinants of stillbirth. Addressing these challenges requires early identification and management of high‐risk pregnancies through enhanced prenatal screening, strengthening antenatal care services, promoting facility‐based deliveries, and equipping healthcare providers with skills to manage complications.

## 1 Background

An infant born either during labor (intrapartum stillbirth) or after fetal demise (antepartum stillbirth) is referred to as a stillbirth. Early fetal death is defined by the International Classification of Diseases (ICD) (ICD 10th and 11th) as the death of a baby born in utero who shows no indications of life at birth [1, 2]. At least 2.6 million stillbirths occur annually worldwide, with low‐ and middle‐income countries accounting for 98% of these cases, 77% of which are in South Asia and sub‐Saharan Africa [3, 4]. There were a staggering 48 million stillbirths worldwide, making stillbirth a major cause of death. Approximately one million babies are stillborn in Africa every year, with at least 300,000 of those deaths occurring during childbirth [4]. Ethiopia, a sub‐Saharan nation, has seen notable gains in maternal and child mortality over the previous 15 years; nevertheless, perinatal mortality is still a major challenge, with the nation still having one of the highest rates of deaths per 1000 live births worldwide [5, 6]. The 2016 Ethiopian Demographic Health Survey revealed that the number of stillbirths in Oromia Regional State was more than in other regions of the country. Oromia had 39 stillbirths per 1000 people, second only to Amhara Regional State's 50/1000 [7].

Major obstetric complications like stillbirth have a lot of severe effects on bereaved parents and siblings [8]. The remarkable journey through months of pregnancy comes to an abrupt and painful end when the baby is born dead. The impact of this event extends beyond the loss of the newborn to include psychological, social, and economic ramifications for parents, families, caregivers, and entire nations. Stillbirth has serious negative effects on one's health and finances. Following a stillbirth, mothers may have psychological repercussions such as anxiety, despair, PTSD, and stigma [9, 10, 11]. Additionally, stillbirth reflects systemic issues in maternal healthcare quality, further exacerbating maternal health problems and mortality.

In 2014, the World Health Assembly approved a goal to eliminate avoidable stillbirths, which was incorporated into the Every Newborn Action Plan and supported by 194 nations, including Ethiopia. The plan's objective was to lower the country's stillbirth rate to 12 or fewer per 1000 live births by 2030 [12, 13]. Maternal, newborn, and child health was prioritized in Ethiopia's national healthcare quality strategy for 2016–2020, which set lofty targets to reduce the country's maternal mortality ratio (MMR) from 412 to 199 per 100,000 live births, its neonatal mortality rate (NMR) from 28 to 10 per 1000 live births, and its stillbirth rate from 18 to 10 per 1000 births by 2020 [14]. However, despite these efforts, stillbirth remains an overlooked issue in many regions of Ethiopia. This study is particularly important as it investigates determinants of stillbirth in public hospitals in the Southwest Shoa zone of Oromia, Ethiopia; in a region where, in 2022 maternal health issues remain critical. Unlike previous studies that were limited in scope, this study addresses the unique sociodemographic, socioeconomic, and cultural context of the study area. By identifying the determinants of stillbirth, this study aims to provide evidence for targeted interventions to improve maternal and perinatal outcomes, addressing gaps in understanding and reinforcing the importance of focusing on maternal health problems in this specific region.

## 2 Methods

### 2.1 Study Design Period and Setting

From September 15, 2022 to October 15, 2022, deliveries attended in public hospitals in the Southwest Shoa Zone were the subject of a hospital‐based, unmatched case‐control research design. One of the Oromia region's 21 administrative zones is the Southwest Shoa Zone. There were 11 woredas in the zone. The capital of the zone is Woliso Town, which is situated 114 km from Ethiopia's capital, Addis Ababa. The zone is home to six public hospitals: St. Lukas Hospital, Bantu Primary Hospital, Amaya Primary Hospital, Leman Primary Hospital, Tullubollo General Hospital, and Woliso General Hospital. Additionally, the population is served by 54 health centers located inside the zone.

### 2.2 Population

The source population consisted of all pregnant women who gave birth in public hospitals in the Southwest Shoa Zone and who were above 28 weeks pregnant.

### 2.3 Study Population

Cases: All women who gave birth to stillbirths in Southwest Shoa Zone public hospitals between July 2020 and June 2022 and whose gestational age was greater than 28 weeks.

Controls: All women who gave birth alive in Southwest Shoa Zone public hospitals between July 2020 and June 2022 and were over 28 weeks of gestation.

### 2.4 Inclusion and Exclusion Criteria

Included were all charts from July 2020 to June 2022 of pregnant women over 28 weeks of gestation who gave birth, both stillborn and alive, in hospitals in the southwest Shoa zone. Mothers' charts lacking information on the main study variables (pregnancy, patient history, labor state, cesarean section, delivery summary, decision note, progress note, and lab results) were disregarded. Mothers' charts that appear missing but were registered in the delivery log book are not included. In a similar vein, the study did not include the pregnant women's charts, which did not indicate if the newborn was alive or dead.

### 2.5 Sample Size Determination

The sample size was determined based on variables previously identified which were determinants of stillbirth, including uterine rupture, maternal hypertension, pre‐eclampsia, absence of prenatal care, and referral as the mode of admission. Given the use of secondary data, careful consideration was given to ensure adequate statistical power and representation. The calculations used Epi Info version 7 with the following assumptions; 95% confidence level; a 1:2 case‐to‐control ratio; 80% power; a 64.7% exposure‐to‐control proportion drawn from the North Shoa Zone Public Hospital study; and an odds ratio of 2.35. Pre‐ and eclampsia were the chosen exposures. The calculated sample size was 288 (96 cases and 192 controls). Adding 10% non‐retrieval rate due to incomplete records, the total sample size was adjusted to 101 cases and 201 controls.

### 2.6 Sampling Procedure

This study included five of the six public hospitals in the Southwest Shoa Zone. The sixth hospital was excluded due to incomplete delivery records for the study period. Delivery logbooks from each hospital were reviewed to compile a list of all neonates born between July 2020 and June 2022. The total number of deliveries (*N* = 17,442) was divided by the total sample size (*n* = 317) to calculate the sampling interval (*K* = 55).

Proportional allocation of cases and controls was done based on the delivery records from each hospital: St. Lukas Hospital: 48 cases, 96 controls; Tullubollo Hospital: 23 cases, 46 control; Leman Hospital: 12 cases, 24 controls; Amaya Hospital: 10 cases, 20 controls; and Bantu Hospital: 8 cases, 16 controls. Cases were selected sequentially from the delivery logbook, while controls were chosen using systematic random sampling at every 55th interval after the first number was randomly selected using a lottery method. Ultimately, 201 controls and 101 cases were chosen.

### 2.7 Operational Definition

Stillbirth is defined as a baby born without any signs of life at or after 28 weeks of gestation [15 ].

Live birth is defined as a baby showing evidence of life (such as the beating of the heart or pulsation of the umbilical cord) on delivery at or after 28 weeks of gestation [1 ].

Early antenatal initiation is the time of antenatal initiation at the second visit or earlier.

Late antenatal initiation is the time of antenatal initiation at the third visit or above.

### 2.8 Data Collection Tools and Procedures

Data from the medical records of study participants born between July 2020 and June 2022 were extracted using a standardized data extraction form. The four components of the English‐designed checklist were for sociodemographic information, information about maternal health and pregnancy, information about labor and delivery, and information about fetal health. The investigator adjusted it after reading several works of literature [16, 17, 18, 19]. In the process of gathering data, the subsequent actions were taken:

Each hospital was first given a single midwifery (BSc) data collector, and from the hospital delivery registration book, all medical record numbers of stillbirths and live births discovered between July 2020 and June 2022 were recognized. Cases are chosen one after the other, while controls are chosen using the *K* (55)th interval from the delivery registration book, which is established in the sample procedure. Data collectors and card room staff used medical record numbers to choose mothers' charts from the card room after choosing cases and controls. The data collectors completed the checklist after reviewing the history, delivery summary, test results, decision notes, and progress notes.

### 2.9 Data Quality Control

The data collectors participated in a 1‐day training session that covered the study's objectives, ethical considerations, and data collection techniques. During this session, they were trained on understanding and accurately extracting information from hospital medical records, including how to use the data collection tool effectively while ensuring patient confidentiality. The study ensured data quality through a robust internal quality assurance process, including training data collectors, daily data verification, double data entry, standardized procedures, supervisory oversight, and comparative validation against similar investigations.

### 2.10 Data Analysis

To confirm the data's validity, the data were cleaned after being entered into the statistical program Epi Info version 7. After exporting the produced data, IBM SPSS version 20 was used to do the analysis. The variance inflation factor (VIF < 10) was used to examine the correlation between the independent variables to verify multicollinearity. The Hosmer and Lemeshow goodness of fit test with a *p*‐value > 0.05 was used to assess the final model's goodness of fit. The study's variables were described using the computation of cross‐tabulation, descriptive statistics like frequency distribution and measures of central tendency (mean and standard deviation), and the assumption of normalcy for continuous outcome variables. 95% Confidence Interval (CI), binary and multivariable logistic regression were employed to determine whether the chosen dependent and independent variables showed any signs of relationship. In the binary logistic regression, 95% CI and *p*‐value < 0.25 were applied to the variables that met the cutoff point. In the multivariate logistic regression model, variables with a *p*‐value < 0.05 were deemed statistically significant factors with stillbirth. Lastly, texts and tables were used to present the findings. The study was conducted after Ethical clearance was obtained from the Ethical Review Board (ERB) of Ambo University, College of Medicine and Health Sciences, and a supporting letter was obtained from the Oromia Regional Health Bureau, which was written to each hospital.

## 3 Results

### 3.1 Sociodemographic Characteristics

The analysis included 302 sampling charts of mothers who gave birth in hospitals located in the southwest Shoa zone, resulting in a 100% response rate. With a standard deviation of ± 5.7 years, the mothers' mean age was 26.4 years. Table 1 shows that 167 (84.1%) controls and 67 (66.3%) cases belonged to the 20–34 age range. Ninety‐one (45.3%) controls and 81 (80%) cases lived in rural areas.

**Table 1:** Sociodemographic characteristics of mothers who gave birth and participated in the study at public hospitals of southwest Shoa zone, Oromia, Ethiopia, 2022.

Variables	Cases	Controls	Total
	*N* (%)	*N* (%)	*N* (%)
Maternal age			
< 20	14 (13.9%)	18 (10%)	32 (10.6%)
20–34	67 (66.3%)	169 (84.1%)	236 (78.1%)
> 34	20 (19.8%)	14 (6.9%)	34 (11.3%)
Residence			
Urban	20 (19.8%)	110 (54.7%)	130 (43%)
Rural	81(80.2%)	91 (45.3%)	172 (57%)
Marital status			
Married	99 (98%)	199 (99%)	298 (98.7%)
Unmarried	2 (2%)	2 (1%)	4 (1.3%)

#### 3.1.1 Maternal Health and Pregnancy‐Related Characteristics

As indicated in Table 2, more than half of cases 52 (51.5%) and controls 111 (55.2%) were in primigravida mothers than others. Most of the cases have occurred in primipara mothers.

**Table 2:** Maternal health and pregnancy‐related characteristics of mothers who gave birth and participated in the study at public hospitals in the southwest Shoa zone, Oromia, Ethiopia, 2022.

Variables	Case	Controls	Total
	*N* (%)	*N* (%)	*N* (%)
Gravidity			
Primi gravida	52 (51.5%)	111 (55.2%)	163 (54%)
Multi gravida	19 (18.8%)	66 (32.8%)	85 (28.1%)
Grand multigravida	30 (29.7%)	24 (11.9%)	54 (17.9%)
Parity			
Primipara	58 (57.4%)	134 (66.6%)	192 (63.6%)
Multipara	29 (28.7%)	61 (30.4%)	90 (29.8%)
Grand multipara	14 (13.9%)	6 (3%)	20 (6.6%)
Stillbirth history			
Yes	11 (10.9%)	12 (6%)	23 (7.6%)
No	90 (89.1%)	189 (94%)	279 (92.4%)
Abortion history			
Yes	7 (6.9%)	28 (13.9%)	35 (11.6%)
No	94 (93.1%)	173 (86.1%)	267 (88.4%)
Preceding birth interval			
≤ 24 months	68 (67.3%)	88 (43.8%)	159 (52.6%)
> 24 months	33 (32.7%)	113 (56.2%)	146 (48.4%)
Other medical illness (Hepatitis B, Rh factors HEELPS, chorioamnionitis)			
Yes	21 (20.8%)	19 (9.5%)	40 (13.2%)
No	80 (79.2%)	182 (90.5%)	62 (86.8%)
Syphilis			
Yes	3 (2.9%)	4 (2%)	7 (2.3%)
No	98 (97.1%)	197 (98%)	295 (97.3%)
HTN			
Yes	3 (3%)	4 (2%)	5 (1.7%)
No	98 (97%)	197 (98%)	297 (98.3%)
Antenatal visit			
Yes	85 (84.2%)	188 (93.5%)	273 (90.4%)
No	16 (15.8%)	13 (6.5%)	29 (9.6%)
Time of antenatal initiation			
Early ANC initiation	74 (73.3%)	180 (89.5%)	254 (84.1%)
Late ANC initiation	27 (26.7%)	21 (10.6%)	48 (15.9%)
Antepartum hemorrhage			
Yes	20 (19.8%)	11 (5.5%)	31 (10.3%)
No	81 (80.2%)	190 (94.5%)	271 (89.7%)
Pre‐eclampsia/eclampsia			
Yes	16 (15.8%)	15 (7.4%)	31 (10.3%)
No	85 (84.2%)	186 (92.5%)	271 (89.7%)
Premature rupture of the membrane			
Yes	30 (29.7%)	57 (28.4%)	87 (28.8%)
No	71 (70.3%)	144 (71.6%)	215 (71.2%)
Hgb level			
< 11 mg/dL	6 (5.9%)	14 (7%)	20 (6.62%)
≥ 11 mg/dL	95 (94.1%)	187 (93%)	281 (93.4%)

### 3.2 Labor and Delivery‐Related Characteristics

Table 3 demonstrates that 47 (28.4%) of the controls and 59 (58.4%) of the patients were referred from other medical facilities for the current delivery. In terms of fetal presentation, aberrant fetal presentation occurred during childbirth in 23 (22.8%) of cases and in 25 (12.4%) of the controls (Table 3).

**Table 3:** Labor and delivery‐related characteristics of mothers who gave birth and participated in the study at public hospitals in the southwest Shoa zone, Oromia, Ethiopia, 2022.

Variables	Cases	Controls	Total
	*N* (%)	*N* (%)	*N* (%)
Mode of admission			
Not referred	42 (41.6%)	154 (76.6%)	196 (64.9%)
Referred	59 (58.4%)	47 (28.4%)	106 (35.1%)
Fetal presentation			
Normal presentation	78 (77.2%)	176 (87.6%)	254 (84.1%)
Malpresentation	23 (22.8%)	25 (12.4%)	48 (15.9%)
Obstructed labor			
Yes	42 (41.6%)	33 (16.4%)	75 (24.8%)
No	59 (58.4%)	168 (83.6%)	227 (75.2%)
Meconium‐stained amniotic fluid			
Yes	22 (21.8%)	35 (17.4%)	57 (18.9%)
No	79 (78.2%)	166 (82.6%)	245 (81.1%)
Duration of labor			
≤ 24 h	53 (52.5%)	176 (87.6%)	229 (75.8%)
> 24 h	48 (47.5%)	25 (12.4%)	73 (24.2%)
Mode of delivery			
Vaginal	71 (70.3%)	130 (64.7%)	201 (66.6%)
C/S	21 (20.8%)	59 (29.3%)	80 (26.5%)
Assisted delivery	9 (8.9%)	12 (6%)	21 (6.9%)
Preterm labor			
Yes	34 (33.6%)	24 (11.9%)	58 (19.2%)
No	67 (66.3%)	177 (88.1%)	244 (80.8%)

### 3.3 Fetal Characteristics

Table 4 shows that 170 (84.6%) controls and 61 (60.4%) cases were delivered between 37 and 41 weeks of gestation. The birth weight range for 62 (61.4%) cases and 169 (84%) controls was between 2500 and 4000 g.

**Table 4:** Fetal‐related determinants of mothers who gave birth and participated in the study at public hospitals of southwest Shoa zone, Oromia, Ethiopia, 2022.

Variables	Cases	Controls	Total
	*N* (%)	*N* (%)	*N* (%)
Gestational age at birth			
< 37 weeks	36 (35.6%)	25 (12.4%)	61 (20.2%)
37–41 weeks	61 (60.4%)	170 (84.6%)	231 (76.5%)
≥ 42 weeks	4 (4%)	6 (3%)	10 (3.3%)
Newborn weight at birth			
< 2.5 kg	35 (34.6%)	25 (12.4%)	60 (19.9%)
2.5–4 kg	62 (61.4%)	169 (84%)	231 (76.5%)
> 4 kg	5 (4.9%)	6 (3%)	11 (3.6%)
Number of newborns			
Single	96 (95%)	188 (93.5%)	285 (94.4%)
Multiple	5 (5%)	13 (6.5%)	17 (5.6%)
Congenital defect			
Yes	18 (17.2%)	9 (4.5%)	27 (8.9%)
No	83 (82.8%)	192 (95.5%)	275 (91.1%)

### 3.4 Determinants of Stillbirth

Crude odds ratios (COR) and 95% confidence intervals (CI) were used in the bivariate logistic regression analysis. To reduce the impact of potential confounding variables, the variables from the bivariate logistic regression analysis with a *p*‐value of less than 0.25 were added to the multivariable logistic regression analysis model. Various maternal medical conditions, pre‐eclampsia/eclampsia, antepartum hemorrhage, referral method of admission, obstructed labor, and labor duration greater than 24 h were all controlled for in multivariable analysis. Congenital defects and newborn gestational age < 37 were found to be factors associated with stillbirth at 95% CI and *p*‐value less than 0.05.

Compared to mothers without any medical conditions, the risks of having a stillbirth were 3.7 times greater for mothers with various medical conditions (AOR: 3.67, 95% CI: 1.45–9.31). Women with pre‐eclampsia/eclampsia had a three fold higher chance of stillbirth than those without these conditions (AOR: 3.33, 95% CI: 1.2–9.2). Furthermore, women who experienced antepartum hemorrhage had a 5.8‐fold increased risk of stillbirth compared to their counterparts (AOR: 5.8, 95% CI: 2.0–15.9). Similarly, the risks of a stillbirth were 2.8 times greater for moms who were referred from another medical facility than for those who were not (AOR: 2.8, 95% CI: 1.4–5.3).

Women diagnosed with obstructed labor had a 3.8‐fold increased risk of stillbirth compared to those without obstructed labor (AOR: 3.8, 95% CI: 1.9–7.8); and those whose labor lasted longer than 24 h had a 4.7‐fold increased risk of stillbirth compared to those whose labor lasted less than 24 h (AOR: 4.7, 95% CI: 2.3–9.6). Compared to their counterparts, mothers with a newborn gestational age of less than 37 weeks had a 3.4‐fold increased risk of stillbirth (AOR: 3.5, 95% CI: 1.6–7.4). Lastly, compared to normal pregnancies, fetuses with birth defects had a seven fold increased risk of stillbirth (AOR: 6.9, CI: 2.4–19.7) (Table 5).

**Table 5:** Bivariate and multivariable logistic regression analysis of determinants of stillbirth among deliveries attended at public hospitals of southwest Shoa zone, Oromia, Ethiopia, 2022.

Variables	Categories	Cases	Controls	COR:	AOR:
		*N* (%)	*N* (%)	95% CI	95% CI
Other medical illnesses					
	**Yes**	**21 (20.8%)**	**19 (9.5%)**	**2.51 (1.28–4.93)**	**3.67 (1.45–9.32)**
	No	80 (79.2%)	182 (90.5%)	1	1
Pre‐eclampsia/eclampsia	**Yes**	**16 (15.8%)**	**15 (7.5%)**	**2.55 (1.10–5.92)**	**3.33 (1.21–9.19)**
	No	85 (81.2%)	186 (92.5%)	1	1
Antepartum hemorrhage	**Yes**	**20 (19.8%)**	**11 (5.5%)**	**6.58 (2.51–17.27)**	**5.84 (2.14–15.93)**
	No	81 (80.2%)	190 (94.5%)	1	1
Mode of admission	Not referred	42 (41.6%)	154 (76.6%)	1	1
	**Referred**	**59 (58.4%)**	**47 (28.4%)**	**4.60 (2.76–7.69)**	**2.77 (1.44–5.31)**
Obstructed labor					
	**Yes**	**42 (41.6%)**	**33 (16.4%)**	**3.62 (2.10–6.24)**	**3.817 (1.87–7.79)**
	No	59 (58.4%)	168 (83.6%)	**1**	**1**
Duration of labor					
	< 24 h	53 (52.5%)	174 (86.6%)	1	1
	**> 24 h**	**48 (47.5%)**	**27 (13.4%)**	**6.38 (3.59–11.31)**	**4.65 (2.26–9.56)**
Newborn gestational age					
	**< 37 weeks**	**36 (35.6%)**	**25 (12.4%)**	**4.01 (2.23–7.23)**	**3.48 (1.63–7.43)**
	37–42 weeks	61(60.4%)	170(84.6%)	1	1
Congenital defect					
	**Yes**	**18 (17.8%)**	**9 (4.5%)**	**5.29 (1.81–15.45)**	**6.94 (2.44–19.73)**
	No	83 (82.2%)	192 (95.5%)	1	1

*Note:* 1 = Reference; Bold = significant variable.

Abbreviations: AOR, adjusted odds ratio; CI, confidence interval; COR, crude odds ratio.

## 4 Discussion

This study found that several critical determinants of stillbirth in public hospitals in the southwest Shoa zone are consistent with broader trends observed in LMICs, particularly in sub‐Saharan Africa. For example, pre‐eclampsia/eclampsia, antepartum hemorrhage, prolonged labor, and obstructed labor are common contributors to maternal and perinatal mortality across the region, as reported in studies from Ethiopia, Ghana, and Nepal [16, 18, 20]. These conditions highlight gaps in emergency obstetric care, referral systems, and prenatal health education, which are persistent in LMICs. Pre‐eclampsia/eclampsia, identified as a significant risk factor, underscores the need for early detection and management through robust antenatal care systems. Many LMICs, including Ethiopia, face challenges in ensuring universal access to quality antenatal care, which limits the early identification and management of hypertensive disorders in pregnancy. This finding is corroborated by research done in Ethiopian public hospitals and in India [16, 21]. This study found that women who experienced antepartum hemorrhage were 5.8 times more likely to give birth to a stillborn child than women who did not. It aligns with research from Hawasa University Hospital, Bale Public Hospital in Ethiopia, and Nepal [18, 20, 22]. The association of stillbirth with gestational age below 37 weeks and congenital abnormalities points to the need for improved neonatal care and maternal nutrition. Preterm births remain a dominant cause of stillbirth and neonatal mortality in LMICs due to limited access to interventions such as corticosteroids for lung maturity and skilled neonatal resuscitation. Integrating nutritional counseling and supplementation into antenatal programs could also help mitigate congenital anomalies, which are exacerbated by micronutrient deficiencies prevalent in LMICs.

This study found that women who experienced antepartum hemorrhage were 5.8 times more likely to give birth to a stillborn child than women who did not. It aligns with research from Hawasa University Hospital, Bale Public Hospital in Ethiopia, and Nepal [18, 20, 22]. This could be because abnormalities in the placenta that cause excessive bleeding, anemia, and poor placental perfusion during pregnancy can cause APH. This can lead to intrauterine hypoxia, infection, and premature delivery, all of which raise the risk of complications.

According to this study, pregnant women who are referred from other healthcare facilities have 2.8 times the chance of having a stillbirth compared to pregnant women who are not. The research carried out at Ethiopia's public hospitals in North Shoa and Bale [16, 18] supports this conclusion. One plausible explanation could be that the majority of research subjects are sent from outlying rural health institutions, and following difficulties (uterine rupture, hemorrhage, and fetal distress), this could lead to a rise in stillbirths. This makes it feasible for rural health facilities to refer patients early, raise knowledge of birth preparation and its complications during prenatal care, and enhance obstetric care and the accessibility of emergency services in remote areas.

Furthermore, the study's findings indicated that women with an official diagnosis of obstructed labor had a stillbirth risk that was 3.8 times higher than that of women without such a diagnosis. The outcomes are consistent with research conducted at Hawasa University Hospital, the public hospital serving the Bale Zone [18, 20]. A probable cause of obstructed labor is when the baby, despite the uterus contracting correctly, is physically blocked and does not escape the pelvis during birthing. This deprives the infant of oxygen, potentially leading to death. This resulted in poor emergency readiness, failure to diagnose at medical institutions, delays in seeking care, or issues with the referral system, which can increase the risk of uterine rupture.

One of the factors in this study that predicts stillbirth is prolonged labor. Mothers whose labor lasted longer than 24 h are 4.7 times more likely to give birth to a stillborn child than mothers whose labor lasted less than 24 h. This result is consistent with research carried out in Hawasa University Hospital, Ethiopia, North Shoa Public Hospital, and northern Ghana [16, 20, 23]. This is because protracted labor results in fetal discomfort and hypoxia, which in turn leads to intrapartum fetal death.

This study indicates that the risk of stillbirth is 3.5 times higher for mothers whose neonatal gestational age was less than 37 weeks. The results of this study are in line with those of other studies carried out in Nepal, Ethiopia, Addis Ababa tertiary hospital, Aksum, and other locations [22, 24, 25]. One cause would be that premature babies are more likely to be asphyxiated and agitated during their brief time in the mother's uterus, ultimately resulting in stillbirth.

Lastly, the current study also showed that there is a 6.9% increase in the probability of stillbirth due to prenatal abnormalities. This outcome is consistent with the research conducted at Ethiopia's Felege‐Hiwot Hospital and North Shoa Public Hospital. Moreover, it bears similarities to the research carried out at the Hawasa University Hospital in Ethiopia [16, 20, 26]. A congenital anomaly is any defect, either structural or functional, that results in intrauterine problems or even death; this includes metabolic disorders. Genetic abnormalities are the source of congenital deformities, and a probable lack of access to adequate nutritional diets during pregnancy may put pregnant women at a higher risk. This study has certain limitations; since it uses secondary data, it does not take into account some of the factors that are addressed in the community, such as wealth index, nutritional status, educational status, and accessibility to healthcare services. Since the study is institutional in nature, its generalizability may be restricted to the community level. As a result, while interpreting the findings, readers should take into account the study's limitations.

## 5 Conclusion

This study offers valuable insights into the multifactorial determinants of stillbirth in public hospitals in the southwest Shoa zone, highlighting antepartum hemorrhage, pre‐eclampsia, obstructed labor, prolonged labor, referral‐related delays, congenital anomalies, and preterm birth as key contributors. Its strength lies in the comprehensive multivariate analysis that captures the interrelated nature of these risk factors. The study underscores congenital abnormalities as the strongest predictor of stillbirth, emphasizing the need for preconception care—a critical, often neglected intervention in LMICs—to address upstream determinants. Additionally, the findings suggest the importance of strengthening referral systems and maternal health services to reduce delays and improve outcomes. By identifying these risk factors, the research contributes to evidence‐based policymaking aimed at reducing stillbirth rates in similar settings, paving the way for future interventional studies focused on preconception care, antenatal care, and maternal nutrition.

## Author Contributions

**Bereket Abebe Alayu:** conceptualization, methodology, software, formal analysis, and writing – original draft. **EliasTeferi Balla:** conceptualization, investigation, validation, visualization, methodology, formal analysis, supervision, and data curation. **Daniel Belema Fekene:** methodology and data curation. **Galana Takele Namara:** data curation and supervision. **Marta Fekadu:** investigation and validation.

## Ethics Statement

The study proposal received ethical approval from the Ambo University College of Medical and Health Sciences ethical review committee. The administrative bodies of the respective district administrations were asked for their permission to conduct research in the area.

## Consent

Informed oral consent was obtained from participants.

## Conflicts of Interest

The authors declare no conflicts of interest.

## Transparency Statement

The lead author EliasTeferi Balla affirms that this manuscript is an honest, accurate, and transparent account of the study being reported; that no important aspects of the study have been omitted; and that any discrepancies from the study as planned (and, if relevant, registered) have been explained.

## Data Availability

On reasonable request, the data sets that support this paper can be acquired from the corresponding author.
